# The Heimlich maneuver and chest compression relieve mask ventilation difficulties caused by asymptomatic laryngeal mass: A case report and review of literature

**DOI:** 10.1097/MD.0000000000036362

**Published:** 2023-12-01

**Authors:** Xuefei Zhou, Longfei Wang, Yonghua Zhang, Qiuyue Wu, Yunfei Cao

**Affiliations:** a Department of Anesthesiology, Beilun District People’s Hospital of Ningbo, Ningbo, China; b Department of Anesthesiology, Qilu Hospital (Qingdao), Cheeloo College of Medicine, Shandong University, Qingdao, China; c Department of Respiratory, Beilun District People’s Hospital of Ningbo, Ningbo, China.

**Keywords:** chest compression, difficult mask ventilation, flexible bronchoscopy, Heimlich maneuver, supraglottic mass

## Abstract

**Rationale:**

Some laryngeal masses are typically asymptomatic and easily ignored. However, they can be rare causes of unanticipated difficult airway, leading to critical situations such as “cannot ventilate” or “cannot ventilate and cannot intubate” during anesthesia induction. Inappropriate airway management in such scenarios can have catastrophic consequences for an anesthetized patient. Here we report a case of sudden, unanticipated difficult mask ventilation caused by an asymptomatic supraglottic mass during sedative induction, which was quickly and effectively relieved by the Heimlich maneuver and chest compression.

**Patient concerns:**

We report a rare case of airway crisis occurred during sedative induction in a 63-year-old patient scheduled for a routine flexible bronchoscopy, and no evidence of respiratory difficulty or signs of obstruction was found in preoperative evaluation.

**Diagnoses:**

A detailed examination of laryngopharyngeal structure under bronchoscopy revealed a supraglottic soft-tissue mass with a size of 1.6 × 0.8 cm covering the membranous part of the glottic area, which was the true cause of difficult mask ventilation in this patient during sedative induction.

**Interventions:**

As the unanticipated difficult mask ventilation occurred, 2-handed mask ventilation was initiated immediately for 9 attempts but failed. Fortunately, the airway crisis was successfully relieved with 2 Heimlich attempts and 3 chest compressions, and no need for a laryngeal mask airway.

**Outcomes:**

Once the airway crisis was relieved and the supraglottic mass was confirmed, the patient underwent a second sedative anesthesia and a successful laryngeal mask airway-assisted bronchoscopy, with no post-bronchoscopy adverse events.

**Lessons:**

Asymptomatic supraglottic masses can cause valve-like upper airway obstruction and lead to unanticipated difficult mask ventilation. The Heimlich maneuver and chest compression may be effective in such critical situations and can serve as an emergency intervention.

## 1. Introduction

Management of difficult airway is always a challenge to anesthesiologists. When anticipated, it allows time and preparation to ensure patient safety, however, in cases of unanticipated and sudden difficult airway, especially in outpatient operating rooms where anesthesiologists and difficult airway tools are usually insufficient, it is often too late to establish an effective artificial airway to maintain adequate oxygenation.^[1,2]^ Both the Heimlich maneuver and chest compression have long been known to solve the problem of foreign body obstruction in the airways by increasing pressure in the lungs and forcing air out of the lungs.^[3,4]^ In this case report, we describe an unanticipated difficult mask ventilation (DMV) during sedative induction in a patient with an asymptomatic laryngeal mass, the Heimlich maneuver and chest compression was promptly performed in this life-threatening situation and successfully relieved the airway crisis.

## 2. Case presentation

A routine checkup conducted 2 days before admission, revealed a space-occupying lesion in the right lung of a 63-year-old male patient. Then, the patient was admitted to our respiratory department for further evaluation, though he had no complaints of chest discomfort, cough, expectoration, gasping, or wheezing upon admission. He also denied having a history of hypertension, diabetes, prior surgeries, allergies, drug use, smoking, or alcohol consumption. The only respiratory concern reported was mild snoring at night, as mentioned by his daughter. The patient’s personal and family medical history was unremarkable.

The patient was 172 cm tall and 73 kg in weight, with a body mass index of 24.7 kg/m^2^. Preoperative evaluation of the patient showed that Anesthesiologists Physical Status I, mallompati I, and neck joint mobility was not limited. There was no evidence of respiratory difficulty or signs of obstruction, and no abnormality was found in laboratory assessments, including routine blood, urine and feces analyses, hepatonephric function, and blood coagulation tests. Enhanced CT scan revealed a space-occupying lesion in the upper lobe of the right lung, characterized by a clump-shaped hyperdense image measuring approximately 52 × 30 mm, with mild enhancement upon contrast administration. This imaging result failed to identify the nature of the right lung mass, making it difficult to distinguish between local inflammation and a nodule, and in order to further clarify the diagnosis, a routine flexible bronchoscopy was decided.

On the operation table with standard monitoring, the patient’s preoperative saturation of peripheral oxygen (SpO_2_) was 98% on oxygen. One mL of 2% lidocaine solution was dripped into each nostril, and then a sedation regimen combined with midazolam (3.8 mg, 0.05 mg/kg) and alfentanil (1100 μg, 20 μg/kg), was initiated and administered intravenously. After sedative induction, the Ramsay sedation grade reached 6 (no response to commands or stimuli), and no perceptible chest movements were observed. His SpO_2_ gradually declined below 92%, then 2 anesthesiologists attempted 2-handed pressurized mask ventilation immediately. Quite unexpectedly, though the face mask fit well to the patient’s face without any leaks, a “cannot ventilate” situation occurred with no perceptible chest movements and undetectable end-tidal CO_2_. Despite as many as 9 attempts, both anesthesiologists were unable to administer effective mask ventilation, the patient became cyanotic, and his oxygen saturation decreased at a minimum of 47%. Just as the anesthesiologists were preparing for a LMA insertion, the respiratory physician who was ready to perform the bronchoscopy on the side, attempted 2 Heimlich maneuvers, followed by 3 chest compressions. Remarkably, these interventions immediately relieved the airway crisis, mask ventilation became manageable and relaxed, and within about 15 seconds, the SpO_2_ improved rapidly to 94%, and the patient’s skin color returned to normal. So the LMA insertion was abandoned. After the administration of 0.5 mg of Flumazenil for antagonization, the patient regained consciousness, with his Ramsay sedation grade decreasing to 4 (asleep but responsive to commands), he breathed spontaneously and did not complain of any discomfort.

**Figure 1.**
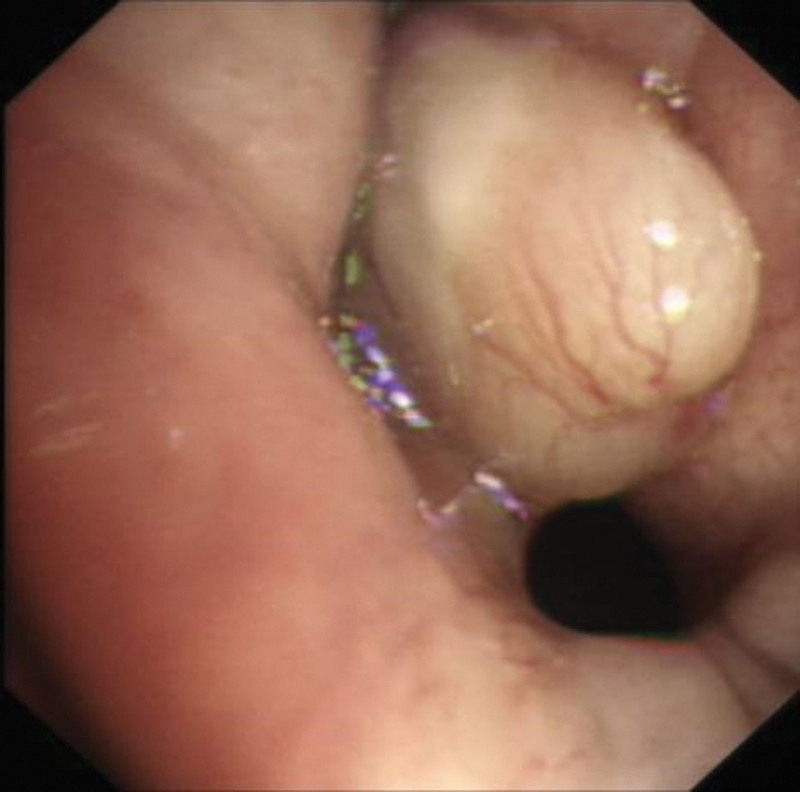
Awake FB demonstrated clear view of the laryngeal mass.

Though the airway crisis was relieved, the cause was unknown. It is possible to identify the problem by examining the laryngopharyngeal structure with a readily available bronchoscope. After consulting the patient and his family and obtaining written informed consent, an awake bronchoscopy quickly revealed a supraglottic soft-tissue mass with a size of 1.6 × 0.8 cm (Fig. 1). Visually, it appeared as a milky white cystic mass with a thin wall, smooth surface, and visible vascular lines. This mass obstructed the membranous part of the glottic area, with its root remaining invisible.

**Figure 2.**
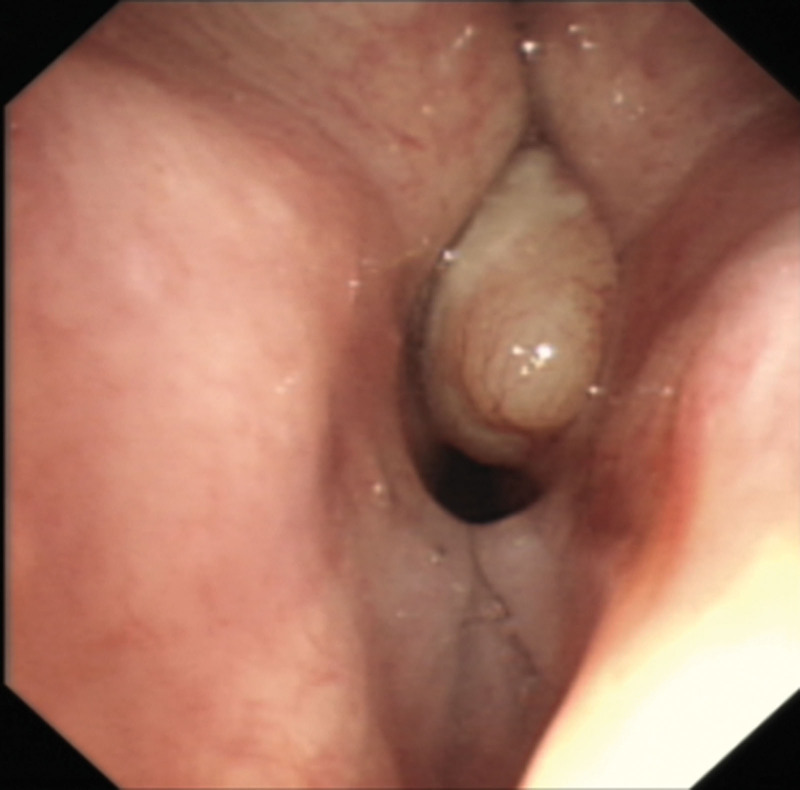
Laryngeal mass after second sedation and LMA insertion.

As the true cause of DMV now identified, a second sedative anesthesia (100 mg propofol and 500 µg alfentanil) was administered intravenously, with LMA insertion and assisted ventilation. A flexible bronchoscopy was performed and revealed a well contraposition of LMA and glottis (Fig. 2). The scope successfully passed the partially exposed glottis into trachea and accomplished routine inspection, including alveolar lavage and bronchial brushing except biopsy, and no tumor cells were detected in the specimens obtained from bronchoalveolar lavage fluid and brush biopsy.

The FB procedure lasted for 11 minutes, and oxygen saturation remained above 94% throughout the procedure. The LMA was extubated once the patient recovered (6 minutes after FB completion). Postoperatively, the patient exhibited stable vital signs without any respiratory complications. The following day, he reported no discomfort and required for discharge, and also refused further examination or treatment for the supragglottic mass.

One year follow-up after discharge, the patient remained asymptomatic, with no respiratory complaints, signs of airway obstruction, or dyspnea/wheezing during spontaneous breathing. However, snoring at night still persisted. The patient still refused further examination of the supraglottic mass, such as CT, MRI, or pathological biopsy.

## 3. Discussion

The Heimlich maneuver is widely recognized by the public as a solution for foreign body airway obstruction.^[3]^ However, it is seldom recommended in many difficult airway guidelines or expert consensus. Even in cases of anesthetized or unconscious patients experiencing DMV, most guidelines and expert consensus typically advocate seeking help, eliminating potential causes of airway restriction or obstruction promptly, and optimizing facemask ventilation.^[1,2]^ In situations when these noninvasive approaches prove ineffective, just like the airway crisis in this case, it is often necessary to establish an artificial airway quickly. According to the 2022 American Society of Anesthesiology Practice Guidelines for management of the difficult airway, when 2-handed pressurized mask ventilation fails to maintain adequate oxygenation, mask ventilation failure should be promptly recognized. In such cases, Supraglottic Ventilatory Devices insertion or optimal intubation attempt should be immediately executed to restore patient’s airway patency and maintain adequate oxygenation and ventilation.^[1,5]^ The Heimlich maneuver and chest compression are not specifically mentioned in difficult airway guidelines, but undoubtedly they are also emergency interventions to eliminate potential causes of airway restriction or obstruction, not only for foreign body blockage of the airway, but also for valvular obstruction caused by an asymptomatic laryngeal mass, as reported in this case. In addition, the Heimlich maneuver and chest compression only need to be performed by hand, do not require other auxiliary devices, and will not affect or delay other interventions.

Considering that difficult airway is always a possibility, routine preoperative airway assessment is essential and usually not difficult for trained anesthesiologists.^[5,6]^ However, it must be noted that even for patients with no signs of difficult airway evaluated preoperatively, there is still the possibility of difficult airway, such as in this case, an unanticipated, abrupt DMV occurred during sedative induction.

Several reports have described airway obstruction due to various types of laryngeal masses during anesthetic induction, including sedation, mask ventilation, and tracheal intubation.^[7–9]^ It is crucial to recognize that even in a patient with relatively small laryngeal masses that do not completely obstruct the airway and show no preoperative airway resistance, severe airway obstruction can occur during anesthetic induction. In this case, Our anesthesiologists were initially overconfident in mask ventilation, and did not realize that a rare DMV had occurred, until alternate attempts at 2-handed pressurized mask ventilation failed. By the time DMV was recognized, the situation had become critical. Fortunately, the respiratory physician attempted urgently the Heimlich maneuver followed by chest compression, and effectively relieved the airway crisis. For a respiratory physician with more than 20 years of clinical experience, these first aid techniques were relatively easy to perform. Relevant literature showed that the Heimlich maneuver can produce a flow of 940 cm^3^ of air in 1/4 seconds (a flow rate of 205 l/minute) by pressing the diaphragm upward, and chest compression can also produce similar intrapulmonary pressure.^[4]^ However, a randomized crossover design study by Langhelle A et al^[3]^ simulating complete airway obstruction on 12 unselected cadavers showed that the mean peak airway pressure was significantly lower with abdominal thrusts compared to chest compressions (26.4 ± 19.8 cm H_2_O vs 40.8 ± 16.4 cm H_2_O), so they believe, chest compression may have the potential of being more effective than Heimlich maneuver for the management of complete airway obstruction by a foreign body in an unconscious patient, though more research is needed to confirm this issue.

Our patient did not complain any discomfort in daily life, such as gasp, suffocate, hoarseness, pharyngeal pain, or a sensation of a foreign object in the throat. However, during sedative induction, a “cannot ventilate” situation occurred. This can be attributed to the fact that the laryngopharyngeal muscles, which were previously maintaining the soft-tissue mass away from the glottis, lost their muscle tone due to sedoanalgesic drugs, allowing the mass to fall into place, and completely obstructing the glottis.^[5,10]^ Numerous studies support the view that opioids, benzodiazepines, other anesthetic induction agents, and muscle relaxants reduce laryngopharyngeal muscle tone, resulting in potential risks of upper airway narrowing and collapse.^[5,6,11]^ In this case, we administered sedative induction without muscle relaxants, and the reduced laryngopharyngeal muscle tone still caused the mass to fall, resulting in a valve-like airway obstruction, as confirmed by a subsequent awake bronchoscopy.

We speculated that the Heimlich maneuver and chest compression increased intrathoracic pressure, thereby creating and sustaining higher airway pressures, which in turn, facilitated the rapid removal of a severe valve-like airway obstruction. However, the application of the Heimlich maneuver and chest compression in the context of difficult airway, including DMV, has not been widely reported in the literature.

## 4. Conclusion

In conclusion, an asymptomatic supraglottic mass can lead to unanticipated DMV during anesthesia induction. The Heimlich maneuver and chest compression may serve as valuable emergency alternatives for rapidly relieving DMV in such critical situations, but more clinical cases are needed to confirm this.

## Acknowledgments

We are deeply grateful to Songyuan Yu and Qi Ding from Endoscopy Center for their assistance and enthusiastic cooperation during the practice of this case.

## Author contributions

**Conceptualization:** Yunfei Cao.

**Data curation:** Xuefei Zhou, Longfei Wang.

**Funding acquisition:** Qiuyue Wu, Yunfei Cao.

**Project administration:** Yonghua Zhang, Qiuyue Wu.

**Supervision:** Yunfei Cao.

**Writing – original draft:** Xuefei Zhou, Longfei Wang.

**Writing – review & editing:** Yunfei Cao.
